# Analysis of Spatial Variations and Sources of Heavy Metals in Farmland Soils of Beijing Suburbs

**DOI:** 10.1371/journal.pone.0118082

**Published:** 2015-02-06

**Authors:** Jianmei Zou, Wei Dai, Shengxuan Gong, Zeyu Ma

**Affiliations:** College of Forestry, Beijing Forestry University, No. 35, Qing Hua East Road, Hai Dian District, Beijing, 100083, China; Institute for Sustainable Plant Protection, C.N.R., ITALY

## Abstract

To understand the effect of intense human activities in suburbs on environmental quality, we obtained 758 measurements of the heavy metals in certain farmland soils of the Beijing suburbs. Multivariate statistical analysis and geostatistical analysis were used to conduct a basic analysis of the heavy metal concentrations, the distribution characteristics and the sources of pollution of the farmland soils in these suburbs. The results showed the presence of eight heavy metals in the agricultural soils at levels exceeding the background values for As, Cd, Cr, Cu, Hg, Ni, Pb, and Zn. In particular, all the measured Cr concentrations exceeded the background value, while As, Cd, Cr, Cu, Hg, Ni, Pb, and Zn were present at 1.13, 1.68, 1.95, 1.43, 1.63, 0.79, 0.92 and 1.36 times their background values, respectively. The results of correlation, factor and spatial structure analyses showed that Cd, Cu, Pb and Zn were strongly homologous, whereas Cr and Hg showed a degree of heterogeneity. The analysis further indicated that in addition to natural factors, Cd, Cu, Pb and Zn in the soil were mainly associated with distribution from road traffic and land use status. Different agricultural production measures in the various areas were also important factors that affected the spatial distribution of the soil Cr concentration. The major sources of Hg pollution were landfills for industrial waste and urban domestic garbage, while the spatial distribution of As was more likely to be a result of composite pollution. The regional distribution of the heavy metals indicated that except for Cr and Hg, the high heavy metal levels occurred in districts and counties with higher organic matter concentrations, such as the northwestern and southeastern suburbs of Beijing. There was no significant Ni pollution in the agricultural soils of the Beijing suburbs.

## Introduction

The suburbs of cities are urban-rural ecotones with unique geographical locations and various activities, a situation that not only induces industrial development in the region but also makes the region an important vegetable and grain production area. The dual influence of this industrial and agricultural production leads to an increased risk of heavy metal pollution for the regional farmland soils and puts soil quality under tremendous pressure [1]. Therefore, it is extremely important to fully control changes in the heavy metal concentrations of suburban soils and in their spatial distributions as well as to conduct further in-depth analysis of the various sources of heavy metal pollution [2].

Several researchers have performed related studies on the heavy metals of agricultural soils from the suburbs of Beijing [3–5]; however, the studied areas have mostly been concentrated in a certain suburban county or a small sewage-irrigated area [3]. Additionally, only a few studies have provided a statistical description [4] or a single-element analysis [5] of the situation in the suburbs as a whole. In recent decades, several studies have introduced geographic information systems (GIS) technology into the analysis of soil heavy metals and used the ordinary kriging model for data calculation and interpolation. However, because the assumptions of the kriging interpolation model were ignored in these applications, that is, because the ordinary kriging model was used for calculation and interpolation without examining the distribution characteristics of the data, there were certain limitations in the results that may affect the accuracy of the analyses. For these reasons, we studied the farmland soils of several suburbs (Changping, Tongzhou, Shunyi, Daxing and Haidian) that are close to the urban area of Beijing through the survey and analysis of eight heavy metals, As, Cd, Cr, Cu, Hg, Ni, Pb, and Zn in the farmland soils of these regions. After a statistical description and analysis of the spatial distribution and data tendencies, an optimal interpolation model and parameters were selected for each heavy metal for further estimation and in-depth investigation of the spatial structure characteristics to analyze the pollution sources of the heavy metals and scales in the agricultural soils of the Beijing suburbs. The study provided supporting data and scientific evidence for the comprehensive understanding of heavy metal distributions in the farmland soils of the Beijing suburbs and for improving the quality of the suburban soil environment.

## Materials and Method

### Sampling site distribution and sample collection

No specific permissions were required for these locations/activities. I confirm that the field studies did not involve endangered or protected species.

**Fig 1:**
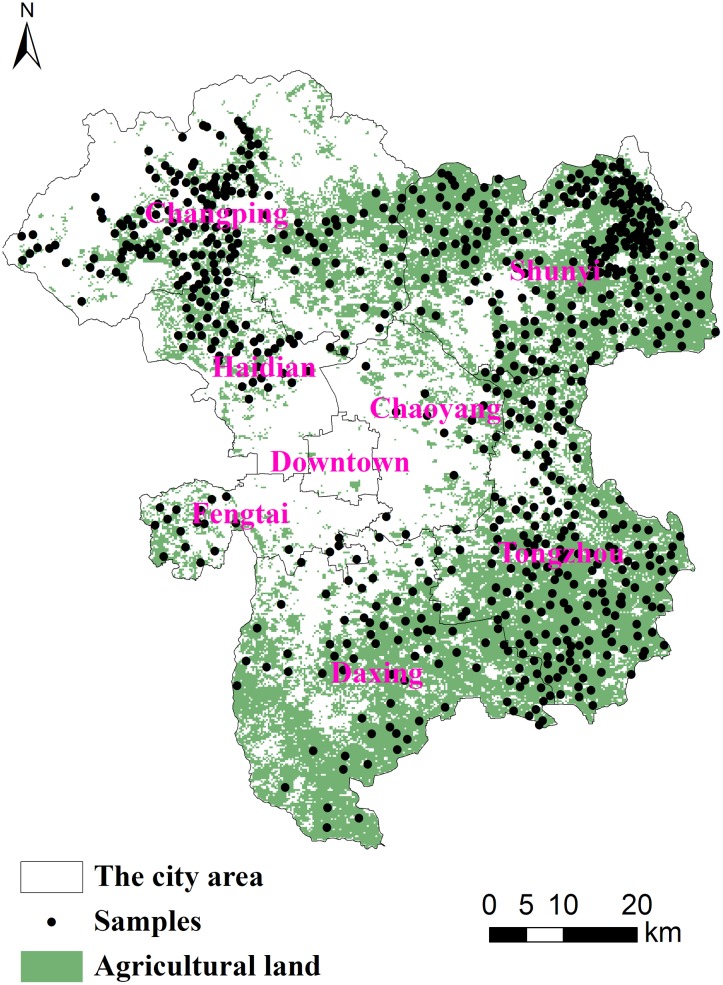
Distribution of sampling sites.

Five counties/districts in the Beijing suburbs, the Changping, Tongzhou, Shunyi, Daxing and Haidian areas, were selected for this study in 2010. A total of 758 sampling (Fig. 1) sites on were collected from those’s suburban farmlands in which the dominant soils are fluvo-aquic soils developed from deposited alluvium, which are classified as Fluvents of Entisols in the United States Department of Agriculture (USDA) Soil Taxonomy. The pH of these sample sites are arranged from 6.5 to 8.5. Most of them are selected from cultivated land, but there is still a few of them picked from the orchard land and grass land.

Approximately A 30- × 30-m sampling area was chosen at each site, and then, 0- to 20-cm mixed soil samples were collected from the area using an S-shaped sampling device. The coordinates of the sampling points were recorded by a global positioning system (GPS) with the georeference coordinates of WGS_84. In addition, the samples were taken only after six consecutive days without rain.

### Sample preparation and analysis methods

The samples were air dried at room temperature, crushed using a wooden stick, passed through a #20 nylon sieve (0.84 mm) and a #100 nylon sieve (0.149 mm) and then stored for measurement. The soil samples were digested in triplicate with aqua regia, one gram dried soil digested with 6 ml aqua regia at 120°C for one hour and at 160°C for 3–4 hours, followed by the addition of 5–6 ml perchloric acid to continue digestion at 160°C for another 5–6 hours until the soil became gray. Then, we were able to use the Foss digestion system.

A heavy metal is originally defined as a metal with a density greater than 5 g/cm^3^, including Ag, Au, Cu, Fe, and Pb. Accumulating heavy metals to a certain level can cause chronic poisoning to the human body. However, no clear definition of heavy metal can be found in the literature. In chemistry, metals are divided into heavy metals and light metals according to their densities: usually, metals with a density greater than 4.5 g/cm^3^ are called heavy metals, which include approximately 45 metals, such as Ag, Au, Cd, Co, Cu Cr, Hg, Ni, Pb and Zn. However, from the perspective of environmental pollution, the definition of heavy metal is extended to Cr, Cd, Hg, Pb and semi-metals (e.g., As) with significant biological toxicity. The environmental definition of heavy metal was adopted in this study. So, the As and Hg concentrations were determined using atomic fluorescence spectrometry (Titan AFS 830, China); the Cu, Cr, Ni, Pb and Zn concentrations were determined using inductively coupled plasma-mass spectrometry (Thermo Icap 6300, USA); and the Cd concentration was determined by atomic absorption spectroscopy (Agilent, AAS, USA). All of the chemicals used in the experiment were guaranteed reagent grade. A standard reference material, environmental standard soil (ESS-1), obtained from the Center for National Standard Reference Material of China, was added during the determination of each heavy metal for quality control. Good quality assurance was achieved from the comparison between the data obtained from this work and the certified values, with recoveries between 93 and 108%. The samples of the soil digests and the blanks were set up in triplicate for analysis, and the analytical error was within 10%.

### Data processing

The Statistical Package for the Social Sciences (SPSS) version 18.0 software (SSPS Inc., Chicago, IL, USA) was used to analyze the statistical characteristics of the data and to examine the normality of their distributions; the Geostatistical Analyst module of the ArcGIS 9.3 software was used to analyze the central spatial tendency. Semivariance analysis software, GS+ version 7 (Gamma Design Software, Plainwell, MI, USA), was used to calculate the semivariogram parameters. Appropriate interpolation models were chosen to perform optimal and unbiased spatial interpolation on the concentrations of the eight heavy metals using the Geostatistical Analyst modules of ArcGIS 9.3 [6–8].

## Results and Analysis

### Analysis of soil heavy metal characteristics

Except for Ni and Pb, the mean concentrations of the studied heavy metals were all higher than their respective average background values. In particular, Cr most often exceeded the standard, with the measurements at all 758 sampling sites surpassing the background value. Moreover, As, Cd, Cr, Cu, Hg, Ni, Pb, and Zn were present at 1.13, 1.68, 1.95, 1.43, 1.63, 0.79, 0.92 and 1.36 times their background values, respectively. The background values selected in this study are from the most authoritative background value reference system (Table 1). The Cd and Hg concentrations in the soils showed greater spatial heterogeneity than the concentrations of the other heavy metals, with coefficients of variation of 0.50 and 0.86, respectively (Table 1). Because the entire study was located on plains areas without significant differences in the types of soil parent material or land use, the strong spatial heterogeneity in the distribution of these two heavy metals was most likely directly related to human activities. The coefficients of variation for the As, Cr, Ni, Pb and Zn soil concentrations were small, indicating a relatively uniform external influence on these metals and the likely homogeneity of their concentrations throughout the region [9].

**Table 1:** Statistics of heavy metal concentrations in agricultural soils of the suburbs of Beijing.

Soil heavy metal	Sample count	Minimum / mg·kg^-1^	Maximum / mg·kg^-1^	Mean / mg·kg^-1^	Standard deviation mg·kg^-1^	Coefficient of variation	Background value mg·kg^-1^	Standard-exceeding rate /%
Cu	758	11.87	97.22	26.78	9.21	0.34	18.7	94.72
As	758	3.51	12.82	7.99	1.31	0.16	7.09	60.55
Cd	758	0.07	1.04	0.20	0.10	0.50	0.119	34.70
Cr	758	36.38	102.96	58.15	6.74	0.12	29.80	100.00
Hg	758	0.01	0.83	0.13	0.11	0.86	0.08	56.20
Ni	758	11.76	31.50	21.22	2.35	0.11	26.80	1.19
Pb	758	4.81	60.78	22.64	6.51	0.29	24.60	28.10
Zn	758	37.34	207.47	78.03	17.56	0.23	57.50	97.10

Note that the background values of Cu, Cr, As, Cd and Pb are from the “Systematic study on background values of soil heavy metals in Beijing” and that of Hg is from the “Environmental Background Values Data Handbook” [26–27].

### Correlation of heavy metals in soil

Correlation can reflect the association between elements and the similarity of their pollution sources. Table 2 shows a significant correlation between the concentrations of Cu and the concentrations of Cd, Pb and Zn. The correlation coefficients were large, between 0.53 and 0.89, and highly significant. As and Ni were moderately but highly significantly correlated. In addition, Cr was moderately correlated with Cd and Zn, whereas Hg was only correlated with As and not with the other heavy metals. These results indicate that there were special sources of the Hg pollution.

**Table 2:** Correlation analysis and coefficients for the heavy metals in agricultural soils.

	Cu	As	Cd	Cr	Hg	Ni	Pb	Zn
Cu	1							
As	0.27**	1						
Cd	0.78**	0.13	1					
Cr	0.12	-0.035	0.26**	1				
Hg	0.046	0.34*	-0.068	0.092	1			
Ni	0.130	0.22**	-0.026	-0.080	-0.087	1		
Pb	0.56**	0.17*	0.51**	0.12	-0.002	0.20**	1	
Zn	0.89**	0.16*	0.82**	0.25**	0.040	0.14	0.53**	1

Note that * and ** denote statistically significant correlation at the 0.05 and 0.01 probability levels, respectively.

### Principal component analysis (PCA) of heavy metals in soils

Based on the results of PCA (Table 3, 4), the eigenvalues of the third extracted components were all greater than 1.0. Thus, the variables could be reduced to a 3 component model that accounts for 71.9% of the variation. Table 3 showed that the first principal component (PC1) explained 40.16% of the total variance and was dominated by Cu, Cd, Pb and Zn (Table 4). The second principal component (PC2) indicated enrichment for As and Hg, and explained an additional 17% of the total variance; PC3 explained an additional 14% of the total variance and was dominated by Cr, Hg and Ni, with a negative loading for Ni. However, the proportions of soil Hg in F2 and F3 were similar. There was no significant correlation between the concentration of Cr or Ni with any of the other six elements, indicating a degree of uniqueness.

**Table 3:** Explanation for the total variance of the factor analysis.

Component	Initial eigen value			Loading extracted sum of squares		
	Total	Variance %	Cumulative %	Total	Variance %	Cumulative %
1	3.213	40.161	40.161	3.213	40.161	40.161
2	1.347	16.839	57.000	1.347	16.839	57.000
3	1.192	14.901	71.901	1.192	14.901	71.901
4	0.853	10.665	82.566			
5	0.582	7.277	89.843			
6	0.523	6.539	96.382			

**Table 4:** All explained variables and factors derived using the orthogonal varimax rotation method.

Element	Before rotation			After rotation		
	F1	F2	F3	F1	F2	F3
Cu	0.924	0.004	-0.040	0.916	0.125	0.021
As	0.307	0.798	0.026	0.199	0.770	0.315
Cd	0.879	-0.239	0.079	0.893	-0.047	-0.191
Cr	0.286	-0.231	0.562	0.266	0.075	-0.612
Hg	0.060	0.666	0.597	-0.074	0.857	-0.252
Ni	0.183	0.382	-0.689	0.188	0.076	0.782
Pb	0.711	0.020	-0.190	0.716	0.044	0.166
Zn	0.931	-0.103	0.026	0.932	0.059	-0.085

### Analysis of spatial index and variability of soil heavy metals

**Normal distribution test, trend analysis and interpolation model selection**. The untransformed and transformed data were tested for conformity to a normal distribution using the SPSS descriptive statistical analysis module and a value of 3 for kurtosis and 0 for skewness. The results showed that except for the raw Ni data and the logarithmic conversion of the Hg data, the data sets did not follow a normal distribution (Table 5).

**Table 5:** Statistical distribution of heavy metals in soils and optimal statistical model analysis.

Element	Untransformed		Logarithmic transformation		Normal distribution status	Central tendency	Optimal interpolation model
	Skewness	Kurtosis	Skewness	Kurtosis			
Cu	4.965	31.914	2.094	8.320	Right-skewed	Exist	Universal kriging
As	-0.203	3.772	-1.000	2.016	Left-skewed	Not exist	Disjunctive kriging
Cd	4.049	23.235	1.177	2.309	Right-skewed	Not exist	Disjunctive kriging
Cr	1.308	4.135	0.445	1.946	Right-skewed	Not exist	Disjunctive kriging
Hg	2.253	6.579	-0.113	3.073	Logarithmic normality	—	Normal kriging
Ni	0.202	4.160	-0.514	1.878	Basic normality	—	Normal kriging
Pb	2.295	10.890	-0.209	6.809	Do not follow	Exist	Universal kriging
Zn	3.419	16.664	1.547	5.531	Do not follow	Exist	Universal kriging

Note because the normality test results for the exponentially transformed data are consistent with those of the raw data, only the parameters for the untransformed and logarithmically transformed data are listed in the table.

According to geostatistical principles, if the raw data or the logarithmic or exponential transformation of the data follow a normal distribution, the ordinary kriging model can be used; if the data are not normally distributed but show a central tendency, the universal kriging model is suitable; and if the data are not normally distributed and show no central tendency, the disjunctive kriging model is used [10]. The dominant trend of the soil concentrations of heavy metals occurs mainly because these concentrations are constrained by soil properties, such as soil texture and geological structure. In this paper, the geostatistical module in ArcGIS was used to select the appropriate interpolation model to determine various factor indicators by considering the distribution characteristics of the heavy metals in soil after analyzing the tendency effect (Table 5).

**Semivariogram**. The spatial variability of the soil heavy metal pollution was described using semivariogram methodology, which reflects the variations between two observed values at different distances [11, 12]. The variable range is the scale of the spatial dependence of the variables, reflecting the distance between the sampling scale and the scale of the factors that affect soil properties [7]. These variables were autocorrelated within the variable range of the sampling distance. Table 6 presents the fitted optimal semivariogram theoretical model and its associated parameters, obtained by fitting the concentrations of the eight heavy metals in the tilled soils of the Beijing suburbs using the GS+ semivariance analysis software. The table shows that the C0/ (C0 + C) values of the six heavy metals other than Ni and Cr were less than 25% and thus exhibited strong spatial correlation. The C0/ (C0 + C) value for Ni and Cr were 50% and 30.4%, respectively, indicating moderate spatial correlation.

**Table 6:** Theoretical semivariogram models for soil heavy metal concentrations and the corresponding parameters.

Element	Theoretical model	Nugget (Co)	Sill (Co + C)	Range (R)	Co/(Co + C)	Coefficient of determination (r^2^)
Cu	Gaussian	27.20	170.70	70,719	0.139	0.999
As	Exponential	0.99	3.992	277,920	0.248	0.932
Cd	Gaussian	0.0028	0.0171	52,810	0.166	0.998
Cr	Gaussian	31.70	104.40	90,707	0.304	0.943
Hg	Exponential	0.0027	0.0132	5,450	0.201	0.996
Ni	Exponential	3.20	6.40	24,810	0.50	0.900
Pb	Gaussian	7.10	85.20	68,086	0.082	0.926
Zn	Gaussian	123.00	656.90	84,870	0.187	0.987

### Spatial distribution of heavy metals in soils

**Fig 2:**
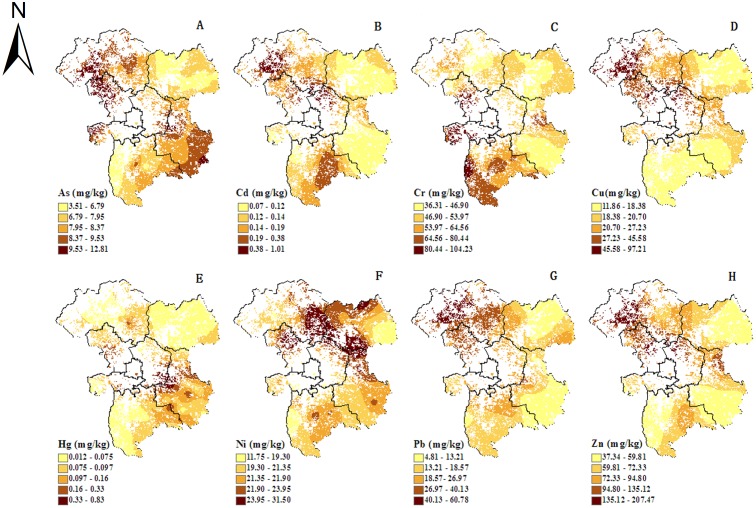
Spatial distribution maps of soil heavy metal concentrations (A–G).

The interpolated spatial distribution of the eight heavy metal concentrations (Fig. 2) plotted with the optimal interpolation model (Table 5), the parameters of the semivariance function model (Table 6) and the corresponding test accuracy (Table 7) show that the peak distribution was in the northwest for Cu, Cd, Pb and Zn; in the southeast for As; mainly on the urban fringe for Hg; and mainly in the southwest for Cr and Cd.

**Table 7:** Cross-validation results of kriging interpolation for soil heavy metal concentrations.

Element	Standardized mean	Root mean square	Average mean error	Standardized root mean square
Cu	0.001057	8.547	8.419	0.9661
As	-0.01437	0.9532	0.9492	1.075
Cd	0.0085	0.097	0.0846	1.042
Cr	-0.002918	6.824	6.404	0.9812
Hg	0.0002034	0.08703	0.1037	0.8104
Ni	0.00718	2.398	1.822	1.228
Pb	-0.002731	4.271	3.327	1.263
Zn	-0.004927	16.87	13.60	1.081

## Discussion

**Fig 3:**
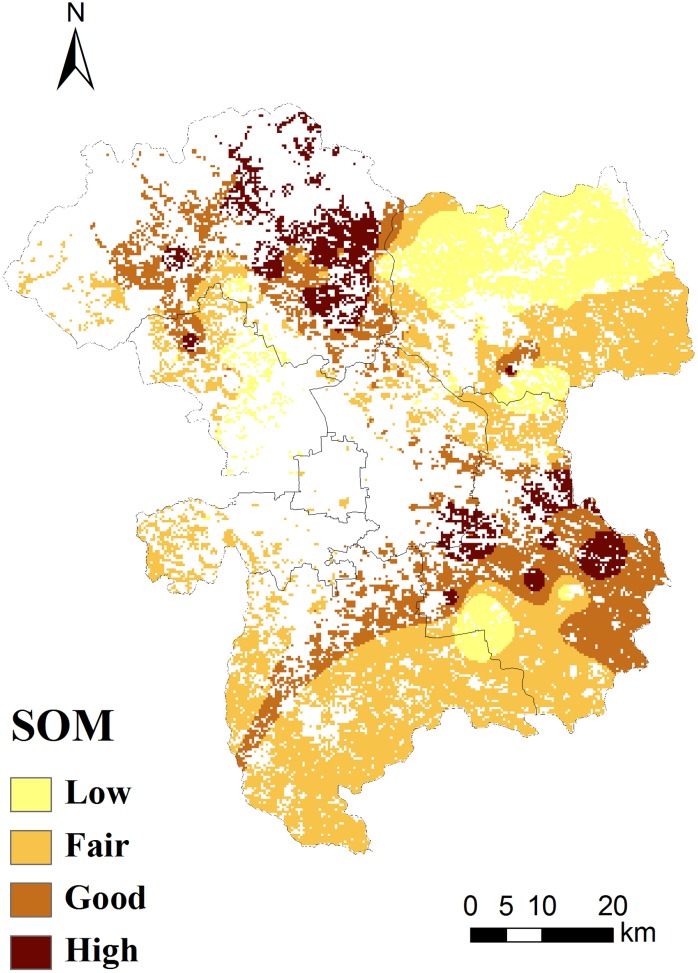
Spatial distribution patterns of SOM estimated by ordinary kriging.

Correlation analysis, principal component analysis and spatial distribution analysis of heavy metals in soil can at least partially reflect the source of the metals. In this study, the combination of these three analyses showed that Cu, Cd, Pb and Zn were strongly correlated and that all were represented in the first principal component (Tab. 3, 4), indicating significant homology. In addition, these four heavy metals, Cu, Cd, Pb and Zn, had high positive loads (0.924, 0.879, 0.711, 0.931), which further indicates that the degree of pollution with these metals was affected not only by the intrinsic properties of soils but also, more importantly, by human activities. Studies show that the Cu level in farmland soils is affected by agricultural activities, such as fertilization, mulching and water irrigation [13, 14], and the accumulations of Pb and Zn are mainly a result of deposition from vehicle exhaust emissions [15, 16], while Cd in soil may be derived from both agricultural production and vehicle exhaust emissions [17–19]. Because of its unique geographical location of suburban farmlands at rural-urban fringes, the location has a dense and well-developed road network and is also an important vegetable and grain production area for urban residents, with active agricultural activities. Therefore, the distribution of roads and the status of land use have become the primary factors influencing the source of heavy metal pollution by Cu, Cd, Pb and Zn in the fringe-area farmland soils. In addition, the spatial distribution of the soil heavy metals in the Beijing suburbs showed that the northwestern suburbs had the highest concentration of the four heavy metals Cu, Cd, Pb and Zn, followed by the southeastern suburbs. Based the data we got from Beijing Environmental Protection Administration of China, we used the Arc GIS drawing a map (Fig. 3) of the organic matter distribution. From the map, we found that the concentration of organic matter got a higher proportion of distribution in the northwest and southeast of Beijing rural areas. Moreover based on the statistics about the field work results, we have found the fact that fertilizers were used most heavily in the farmland soils of the Changping and Tongzhou districts which is exactly located in the corner of northwest and southeast respectively, and compared with the concentration distributions of the four heavy metals, there is an extremely correlated between the highest concentration of Cu, Cd, Pb and Zn and organic matter distribution. Therefore, it can be preliminarily concluded that the increasing application of soil organic fertilizer has gradually become an important new pollutant source that affects the heavy metal concentration of suburban farmland soils.

The concentration of Cr in the soils showed significant heterogeneity and exceeded the guideline concentration value in all the samples, which is consistent with the overall assessment of agricultural soils in Beijing as reported by Huo et al. [20]. Most previous studies suggested that soil Cr is only slightly affected by human activities and mainly affected by rock weathering and erosion [21]. In recent years, certain researchers have indicated that in addition to natural influences, anthropogenic inputs, such as fertilizers and especially the application of phosphate fertilizer and sewage irrigation, have also become important factors affecting soil Cr accumulation [17–19]. The Beijing survey area was relatively small, and the soils in the sampling sites were developed from the same alluvial parent material. However, because sewage irrigation has been used in the southern region for many years, the current soil Cr concentration was significantly higher in the south than in the northern and central regions. Therefore, different production methodology is most likely an important explanation for the difference in accumulated soil Cr concentration in the suburbs.

**Fig 4:**
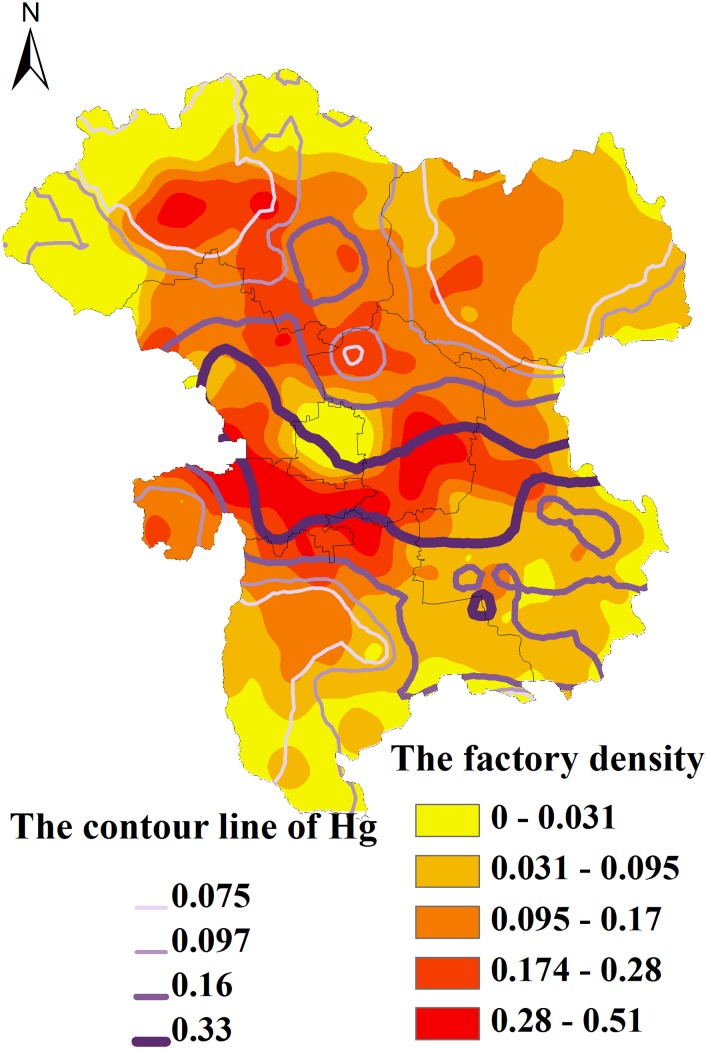
The correlative distribution between the Hg concentrations of spatial contribution and the concentration of the industrial and mining enterprises.

Hg is considered an indicator of industrial and mining waste and of domestic garbage and has the unique property of long-distance propagation [22]. Approximately 6000–7000 tons of atmospheric Hg is produced yearly, and 93.7% of this Hg returns to the soil surface and is absorbed and fixed by clay minerals and organic matter after its long-distance atmospheric dispersal. Nicholson et al. [14] also found that 85% of the Hg in the studied area originated from atmospheric deposition. A distribution map of the industrial and mining enterprises in the Beijing suburbs derived from remotely sensed images (Fig. 4) indicates a high density of industrial and mining enterprises around the city, radiating outward in concentric circles. And we also rate Hg concentration in several levels showing by the contour map, when we overlapped the two maps above, amazing find that there is some similar between them: This concentric concentration of the industrial and mining enterprises basically matched the contour map of the soil Hg concentration distribution (Fig. 4). Therefore, it can be preliminarily determined that there was great variation in the spatial distribution of Hg in the agricultural soils of the Beijing suburbs, and the pollution sources were mainly related to the distribution of industrial and mining enterprises.

Although both the correlation test and the principal component analysis indicated that the As and Hg concentrations in the soils were somewhat homologous, certain studies have indicated that Hg and As can usually be considered indicators of coal and garbage burning [23, 24]. The soil As was mainly from As-containing compounds emitted by industrial and mining production processes [25]. However, the distribution of As in this study did not show significant consistency with the distribution of the industrial and mining enterprises in Beijing. Therefore, the soil As concentration of the study area may be affected by more complex factors.

## Conclusions

The concentrations of the eight heavy metals in the farmland soils from the Beijing suburbs were frequently above the background values, and the soil Cr concentration was the most significant, with Cr > Zn > Cu > Cd > Hg > Pb>Ni.

The concentration distribution of the different heavy metals in the soils had different characteristics. The concentrations of Cu, Cd, Pb and Zn were high mainly in the northwest; in addition to the northwestern region, the As concentration was also significantly increased in the southeast; the peak distribution of Hg was mainly in the urban fringe; and Cr and Cd were high mainly in the south.

The heavy metals accumulated to various degrees in the soils but were from different pollution sources. The Cu, Cd, Pb and Zn pollutant levels were mainly affected by the road distribution and land use status, whereas the application of large amounts of soil organic fertilizer has become an important new factor affecting the heavy metal concentration of the suburban farmland soils. Cr showed significant accumulation in the soils, and in addition to natural factors, different agricultural production measures were implicated as important factors that affected the Cr spatial distribution. Hg and As in the soil showed certain homologies; however, the soil Hg derived mainly from landfills for the waste of industrial and mining enterprises and for domestic waste, whereas the spatial distribution of As was not strongly affected by the disposal of these wastes. Therefore, the As concentration was affected by more complex pollution factors. In addition, there was no significant Ni pollution in the farmland soils of the Beijing suburbs.

And the comparison is based on a mapping of organic fertilization or industrial activities the correlation with element distribution is still just qualitative. we are trying a new spatial estimation method called Mean of Surface with Non-homogeneity (MSN) to find the quantity correlation between them. Maybe in the near future we will hand in the result.

In summary, in recent years, the increased urbanization of the Beijing suburbs, the development of urban-rural industrial and mining enterprises and the increased land use intensity caused by a higher demand-to-production ratio for agricultural lands have affected the ecological environment in this region. Therefore, issues such as the quality of agricultural products, soil pollution and ecological security need to be considered when accelerating the rate of development and construction.

## Supporting Information

S1 Data Complete primary data file. Please see text for explanation of the principal data collected from the samples.
(XLS)
